# Development and validation of a prediction model for recurrence based on ProMisE molecular classifier and immune-inflammatory- nutritional score in endometrial carcinoma: a retrospective multiple-center study

**DOI:** 10.7150/ijms.107134

**Published:** 2025-08-16

**Authors:** Yunfeng Zheng, Fan Yang, Gaohua Liu, Xixi Wu, Langting Xie, Ran Hu, Xiaoxiao Luo, Rui Yuan

**Affiliations:** 1 Department of Gynecologic Oncology, Women's Hospital, School of Medicine, Zhejiang University, Hangzhou, Zhejiang, China; Zhejiang Provincial Key Laboratory of Precision Diagnosis and Therapy for Major Gynecological Diseases, Hangzhou, Zhejiang, China.; 2 Department of Gynecology, The First Affiliated Hospital of Chongqing Medical University, Chongqing, China.; 3 Department of Obstetrics and Gynecology, Renji Hospital, School of Medicine, Chongqing University, Chongqing, China; The Fifth People's Hospital of Chongqing, Chongqing, China.; 4 Cancer Center, Department of Ultrasound Medicine, Zhejiang Provincial People's Hospital, Affiliated People's Hospital of Hangzhou Medical College, Hangzhou, Zhejiang, China.; 5 Institute of Clinical Medicine, The First Affiliated Hospital, Hengyang Medical School, University of South China, Hengyang, Hunan, China.; 6 Department of Gynecology, Chengdu Women's and Children's Central Hospital, School of Medicine, University of Electronic Science and Technology of China, Chengdu, Sichuan, China.; 7 Department of Gynecology and obstetrics, Fujian Provincial Maternity and Children's Hospital, Fuzhou, Fujian, China.; 8 Department of Gynecology and obstetrics, Fujian Medical University Union Hospital, Fuzhou, Fujian, China.

**Keywords:** endometrial carcinoma, molecular classifications, nomogram, HALP score, recurrence-free survival, risk stratification

## Abstract

**Objective**: This study aims to develop a robust prediction model using the ProMisE molecular classification and the prognostic immune-inflammatory-nutritional score to predict recurrence in stage I-III endometrial cancer, thereby enabling risk stratification of high-risk patients.

**Methods**: The clinical data of 582 patients (365 in the training cohort and 217 in the validation cohort) were collected from multiple large cancer centers from patients with stage I-III endometrial cancer who underwent surgical resection between August 2019 and February 2022. Cox proportional hazards regression analysis was used to identify the risk factors for recurrence-free survival (RFS). The concordance index (C-index), area under the receiver operating characteristic (ROC) curves, calibration plots, and decision curve analyses (DCA) were used to assess discrimination and clinical utility of the model.

**Results**: Patients with a hemoglobin, albumin, lymphocyte, and platelet (HALP) score ≤ 31.70 tended to have lower BMI (*P* = 0.017), advanced FIGO stage (*P* = 0.016), deep myometrial invasion (*P* < 0.001), and higher serum Ca125 levels (*P* < 0.001). Multivariate Cox regression analysis showed that age, FIGO stage, grade, LVSI, Ca125, ProMisE molecular subgroup, HALP score, and adjuvant therapy were independent prognostic factors for RFS in patients with endometrial cancer. A nomogram for predicting RFS was established, and patients were stratified into high- and low-risk groups based on the RFS model.

**Conclusions**: The preoperative HALP score serves as a reliable predictor of RFS in endometrial cancer. A nomogram combining the HALP score, ProMisE molecular subtyping, and clinical parameters can assist clinicians in identifying high-risk patients for recurrence. These patients may benefit from early triage and more intensive monitoring.

## Introduction

Endometrial cancer is the most common gynecologic malignancy, with increasing global incidence and mortality rates 1. According to GLOBOCAN 2020 data from the International Agency for Research on Cancer, there were an estimated 63,246 newly diagnosed cases of endometrial cancer and 11,909 cancer-related fatalities in the United States 2. In China, approximately 84,520 new cancer cases and 17,543 cancer deaths are reported annually, posing a serious threat to women's health 3. The majority of patients with endometrial cancer are initially diagnosed with low-grade and early-stage disease, with a favorable prognosis that can be cured through complete surgery alone 4. However, approximately 20% of patients present at an advanced stage or are at high risk of recurrence, significantly contributes to cancer-related deaths 5, 6. Effectively identifying individuals at high risk of recurrence to inform them of their prognosis, perform appropriate surgeries, and administer the best adjuvant therapies to improve their outcomes remains the greatest challenge.

In 1983, Bokhman classified endometrial cancer into type I (estrogen-dependent) and type II (non-estrogen-dependent) based on its clinical and pathological characteristics 7. With the advancement of cancer-related research, the diagnosis and treatment of endometrial cancer are no longer confined to the traditional field of histopathology. The exploration of more precise molecular and immune-inflammatory-nutritional markers for precision therapy has emerged as a prominent focus of current research. The Cancer Genome Atlas (TCGA) molecular classification of endometrial cancer integrates genomic, transcriptomic, proteomic, gene copy number, and methylation data, categorizing endometrial cancer into four subtypes: *POLE* ultra-mutated (*POLE*-mut), microsatellite instability-high (MSI-H), copy number low (CN-L), and copy number high (CN-H) 8. These molecular subgroups, with significant prognostic differences, have been widely validated as effective biomarkers and have been incorporated into the latest guidelines. Due to the requirement for integrating data from multiple platforms and omics disciplines, the clinical application of this molecular classification strategy is quite complex, which limits its widespread adoption is some developing countries or underdeveloped regions. Subsequently, some scholars have further improved and simplified the detection methods and processes for this molecular classification, proposing strategies such as TransPORTEC 9 and ProMisE 10, which have been clinically validated as accurate and effective. These molecular classifications are primarily based on techniques such as immunohistochemistry (IHC) and next-generation sequencing (NGS). These strategies not only simplify the operational and detection processes but also ensure their consistency with the TCGA molecular classification. These clinically relevant molecular subgroups have been replicated using surrogate markers in IHC, identifying equivalent subgroups: p53 abnormalities (p53-abn), *POLE* exonuclease domain mutations (*POLE* EDM), mismatch repair deficiency (MMR-D), and p53 wild-type (p53wt). Integrating this molecular classification with established clinicopathological data has led to an updated risk classification system for determining the relative risk of recurrence.

Emerging evidence demonstrates a significant correlation between systemic inflammation, malnutrition, and the clinical outcomes of cancer patients 11, 12. Several immune-inflammation indices and nutritional scores (i.e. systemic immune-inflammation index 13, 14, neutrophil-to-lymphocyte ratio 15, 16, and prognostic nutritional index 17) have been confirmed as effective biomarkers for prognostic evaluation in various cancers. However, these indicators typically capture only a fraction of the individual's overall status, and the clinical prognosis is often influenced by a variety of complex factors. This underscores the importance of a comprehensive evaluation that encompasses various factors, including systemic inflammation, nutritional status, and immune function. The hemoglobin-albumin-lymphocyte-platelet (HALP) score, as a comprehensive index, effectively reflects the systemic inflammatory response, nutritional status, and immune status. It is utilized to evaluate treatment efficacy and predict the survival outcomes of cancer patients 18, 19. Although the clinical value of molecular classification and the HALP score has been widely recognized and integrated into clinical practice, this classification strategy is still in its early stages in China, especially in the field of endometrial cancer, where widely accepted identification and classification standards have not yet been established. For patients with recurrence or those in advanced stages, combining molecular subtyping with the HALP score for risk assessment and identifying high-risk recurrence populations is of significant clinical importance.

Therefore, we conducted this study across multiple Chinese medical institutions to investigate the value of the HALP score and ProMisE molecular classification in predicting the prognosis of patients with endometrial cancer. A prognostic nomogram was established to assist clinicians in accurately estimating the prognosis.

## Material and Methods

### Patient population

**Figure 1:**
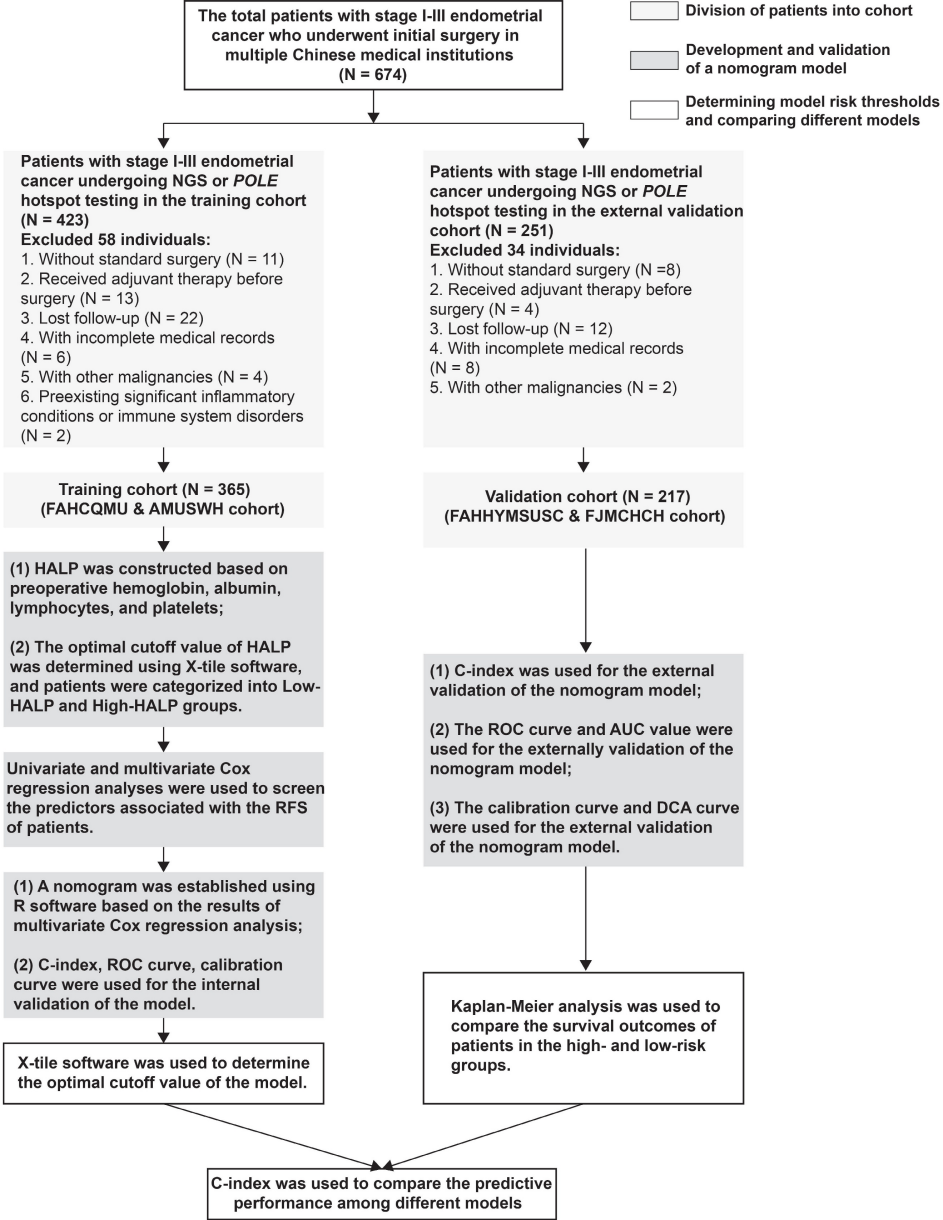
Flow chart for stage I-III endometrial cancer patient inclusion.

Between August 2019 and February 2022, a total of 582 patients with stage I-III endometrial cancer who were treated at multiple Chinese institutions with surgical resection, with or without radiotherapy and chemotherapy, were retrospectively included. Patients who received radiochemotherapy (RChT) prior to surgery and those with incomplete clinicopathological information were excluded from the analysis. Additionally, patients who had concurrent other cancers were also excluded. Details on the inclusion and exclusion criteria, as well as the flowchart of this study, are provided in Figure 1.

### Surgical procedures and postoperative adjuvant treatment

For treatment, all endometrial cancer patients underwent a surgical staging procedure, including total hysterectomy, bilateral salpingo-oophorectomy, and lymph node staging (sentinel lymph node ± pelvic lymph node ± para-aortic lymphadenectomy). Based on comprehensive surgery and pathological staging, radiotherapy was administered within 12 weeks post-operatively, consisting of vaginal brachytherapy (range: 22.0-24.0 Gy, administered in 4 fractions of 5.5-6.0 Gy) and/or pelvic external beam radiotherapy (range: 45.0-50.0 Gy, administered in 25 fractions of 1.8-2.0 Gy) 20. Platinum-based chemotherapy was administered every 3 weeks for 6-8 cycles, either concomitantly or following radiotherapy, based on the assessment by the medical oncologist, considering comorbidities and patient performance status 20.

### Outcomes

The primary endpoint of the study was recurrence-free survival (RFS). All time-to-event data were censored at the last follow-up if the corresponding event had not occurred. All patients who underwent complete staging surgery with lymph node dissection received regular follow-up. Data from patients without recurrence at the last follow-up (August 12, 2024) were censored.

### DNA extraction and sequencing

For specific methods on DNA extraction and sequencing for polymerase epsilon exonuclease domain mutations, please refer to these studies 10, 21. For more information, please consult the previous important research 22, 23.

### HALP score construction and variables

Clinical pathological and follow-up data were systematically gathered by trained assistants using standardized data collection forms, and subsequently verified by a senior physician. Blood test results obtained within one week before the operation were collected, including hemoglobin level, albumin level, lymphocyte count, and platelet count. These blood tests were performed using XE5000 and XE-2100 blood cell counters and reagents (SMK). The HALP value was calculated from the routine blood test results of hemoglobin, albumin, lymphocytes, and platelets (HALP = hemoglobin [g/L] × albumin [g/L] × lymphocytes [/L]/plateltes [/L]) 18.

**Figure 2:**
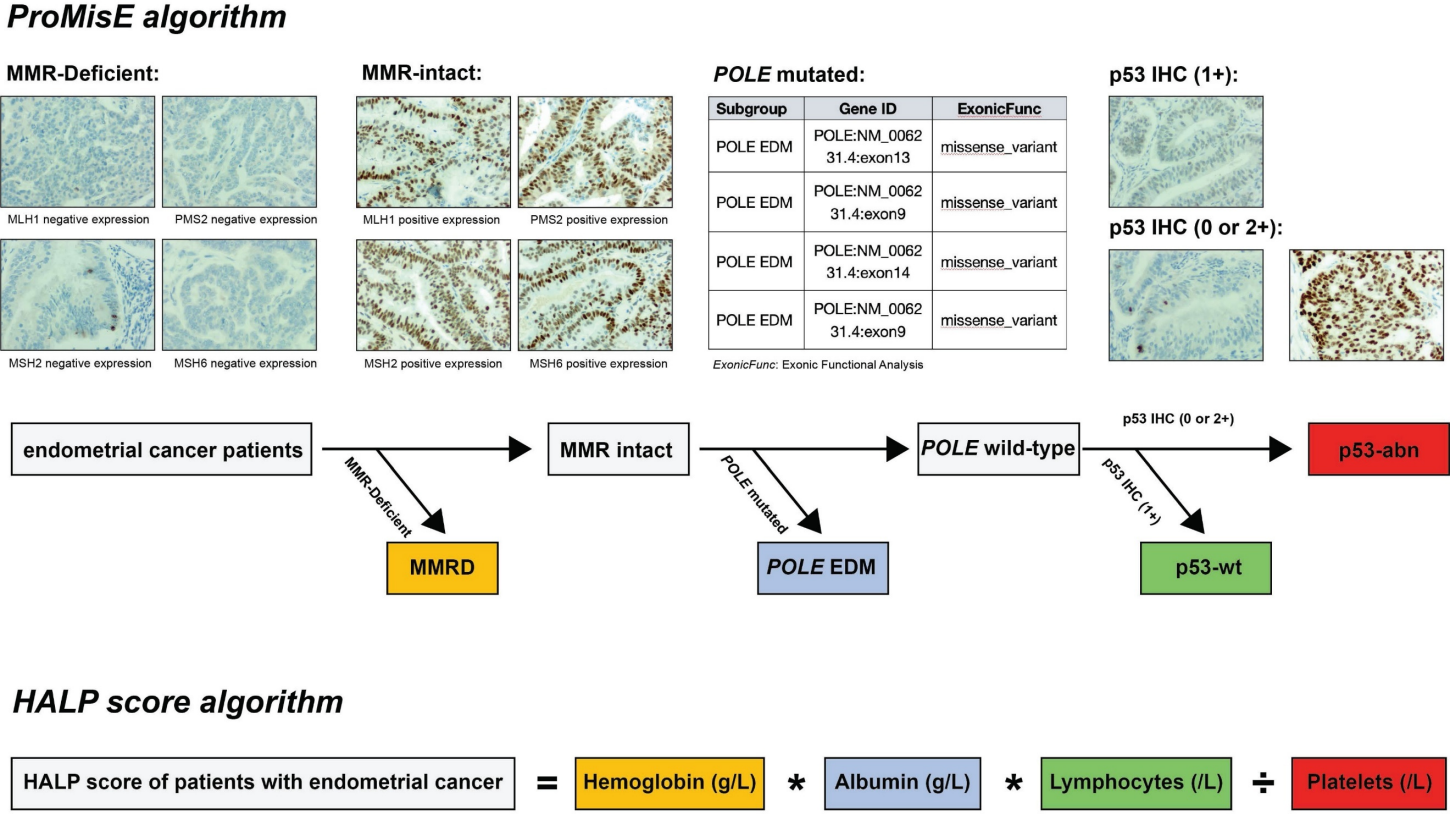
Identification of ProMisE molecular subtypes and calculation of HALP score. Note: ProMisE algorithm: initially, the IHC of MMR proteins (MLH1, MSH2, MSH6, and PMS2) was evaluated, with adequate positive staining in inflammatory cells or stroma as a reference. Complete absence of MMR proteins staining in tumor cell nuclei was regarded as loss of expression, and the absence of any MMR proteins was defined as MMR deficiency (MMR-D); if the MMR proteins were complete expression (MMR intact), further sequencing of tumors was conducted to identify exonuclease domain mutations in *POLE* gene. Mutations in the exonuclease domain of *POLE* were classified as *POLE* EDM (corresponding to the *POLE* ultra-mutated subtype); otherwise, p53 immunostaining should be performed, with complete negativity staining (null) or strong/diffuse staining in > 70% of cells indicated as p53 abnormal (aberrant positive expression, p53-abn), while other staining patterns are classified as wild type (p53-wt). HALP score algorithm: HALP score = hemoglobin [g/L] x albumin [g/L] x lymphocytes [/L]/platelets [/L].

Clinicopathological data encompassed patient age, tumor grade (categorized as grade1/2 or grade 3), FIGO stage (classified as stage I to III), histological type (categorized as endometrioid carcinoma or non-endometrioid carcinoma), depth of myometrial invasion (categorized as ≤ 1/2 or > 1/2), LVSI (categorized as negative or positive LVSI), and ProMisE molecular classification (classified as p53wt, MMR-D, *POLE* EDM, and p53abn). A small proportion of patients harbored multiple molecular features, such as both a *POLE* EDM and p53abn, or MMR-D along with p53abn. However, the ProMisE decision tree determines the sequence for assigning tumors to a specific molecular subtype, categorizing the former example as *POLE-*mut and the latter as MMR-D (Figure 2).

### Statistical Analysis

Continuous variables were expressed as mean ± standard deviation and compared using Student's t-tests (for comparing the means of two continuous variables); categorical variables were presented as frequencies and percentages, and their associations were analyzed using Chi-square (χ^2^) tests or Fisher's exact test. Kaplan-Meier analysis was performed to estimate the survival rates of RFS, and further compared with Mantel-Cox (log-rank) tests. Multiple prognostic factors were collected and included, and identified as independent prognostic factor based on the Cox proportional hazards model. To enhance the reliability of the nomogram, internal validation was conducted using bootstrapping with 1,000 resamples. The model's predictive performance was evaluated through Harrell's concordance index. Additionally, an external independent cohort was utilized to further validate the predictive model. Calibration curves were generated to assess the model's calibration accuracy. Furthermore, decision curve analysis was conducted to measure net benefits at different probability thresholds, offering insights into the nomogram model's clinical utility. Detailed R code is provided in Supplementary Materials R.

All statistical analysis were done using SPSS statistical software (version 21.0, Chicago, IL, USA) and R studio (version 1.2.5019). And the *P*-value of less than 0.05 was considered to indicate statistical significance.

## Results

### Characteristics of the study population

A total of 674 patients with endometrial cancer were included in this study. After excluding 92 patients due to missing and/or uninterpretable follow-up data or clinicopathological data, 582 patients remained for the analysis. Baseline characteristics are presented in Table 1. In the entire study cohort, the mean age of the endometrial cancer patients was 54.26 ± 9.719 years, with a mean BMI of 24.10 ± 3.515 kg/m^2^. Additionally, 84.9% of the patients were diagnosed with endometrioid endometrial carcinoma, followed by non-endometrioid endometrial carcinoma (15.1%). Most patients (66.8%) had negative LVSI, and lower serum Ca125 levels (74.1%). In terms of ProMisE molecular classification, 257 (44.2%) patients harbored p53wt, 148 (25.4%) had MMR-D, 118 (20.3%) had p53abn, and only 59 (10.1%) had *POLE* EDM. The RFS time for the entire cohort was 36.22 ± 9.964 months. Patients in two cohorts had similar clinicopathological features (*P* > 0.05).

### Correlation between HALP score and clinicopathological variables

HALP, as an important indicator reflecting patients' inflammatory, immune, and nutritional status, is closely associated with the prognosis of endometrial cancer patients. The preoperative blood test results obtained within one week before the surgery were collected and are provided in Table S1. In the patient cohort for the present study, we used X-tile software to analyze and determine that the optimal cutoff value of HALP score for predicting recurrence of endometrial cancer is 31.70 (Figure S1). Based on the definition of the cutoff value, the HALP group was divided into a high-HALP group (HALP score > 31.70) and a low-HALP group (HALP score ≤ 31.70). Significant differences in RFS were observed in terms of HALP score, and a low HALP score significantly predicted the dismal RFS in endometrial cancer patients (*P* < 0.001, Figure S2). Additionally, it was found that a lower HALP was significantly correlated with a lower BMI (*P* = 0.017), advanced FIGO stage (*P* = 0.016), deep myometrial invasion (*P* < 0.001), and higher serum Ca125 (*P* < 0.001), but showed no correlation with other clinicopathological variables in the training cohort, which was similar to the external validation cohort (Table 2).

### Establishment of nomogram

**Figure 3:**
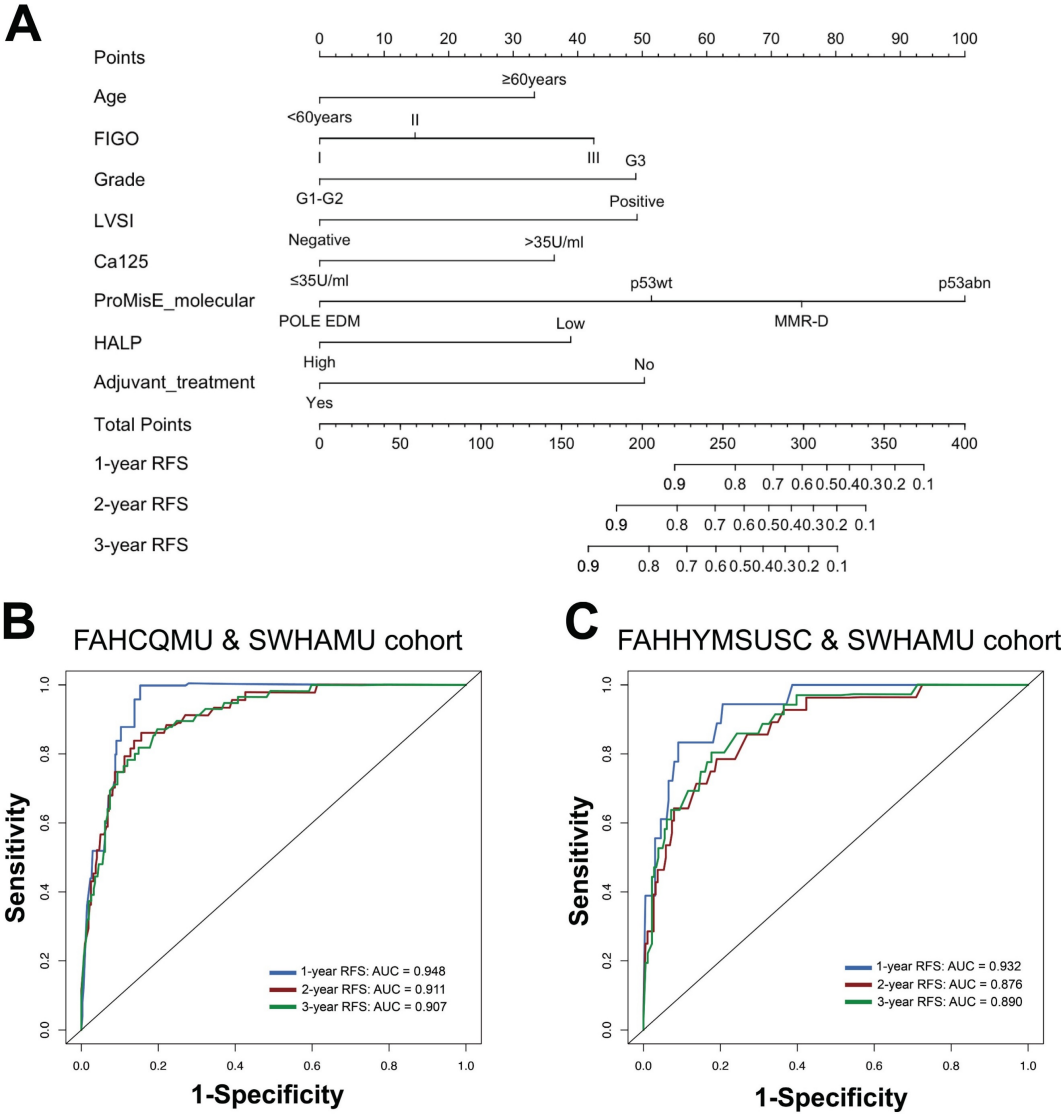
Characteristics in the nomogram model for predicting the probabilities of 1-, 2-, and 3-year RFS in patients with stage I-III endometrial cancer (A); ROC curves of the 1-, 2-, and 3-year RFS rates predicted by the model in both the training cohort (B) and the validation cohort (C).

Univariate and multivariate Cox regression analyses were performed to identify independent prognostic factors. The eight predictors identified by the multivariate regression model were further recruited and used to develop the RFS nomogram, including age [hazard ratio (HR) 1.820, 95% confidence interval (CI) 1.005-3.293], FIGO stage (HR 2.309, 95% CI 1.033-5.164), tumor grade (HR 2.329, 95% CI 1.082-5.016), LVSI (HR 2.304, 95% CI 1.022-5.195), Ca125 (HR 2.100, 95% CI 1.135-3.885), ProMisE molecular classification (HR 2.551, 95% CI 1.093-5.954), HALP score (HR 2.099, 95% CI 1.182-3.728), and adjuvant treatment (HR 0.352, 95% CI 0.160-0.774) (all *P* < 0.05, Table 3). Based on the degree of contribution of each predictor to the resulting events (RFS), corresponding points (the first axis) were determined. Subsequently, the points of each predictor were aggregated to predict the RFS probability of endometrial cancer patients in stage I-III (Figure 3A and Table S2).

### Internal and external validation of nomogram

**Figure 4:**
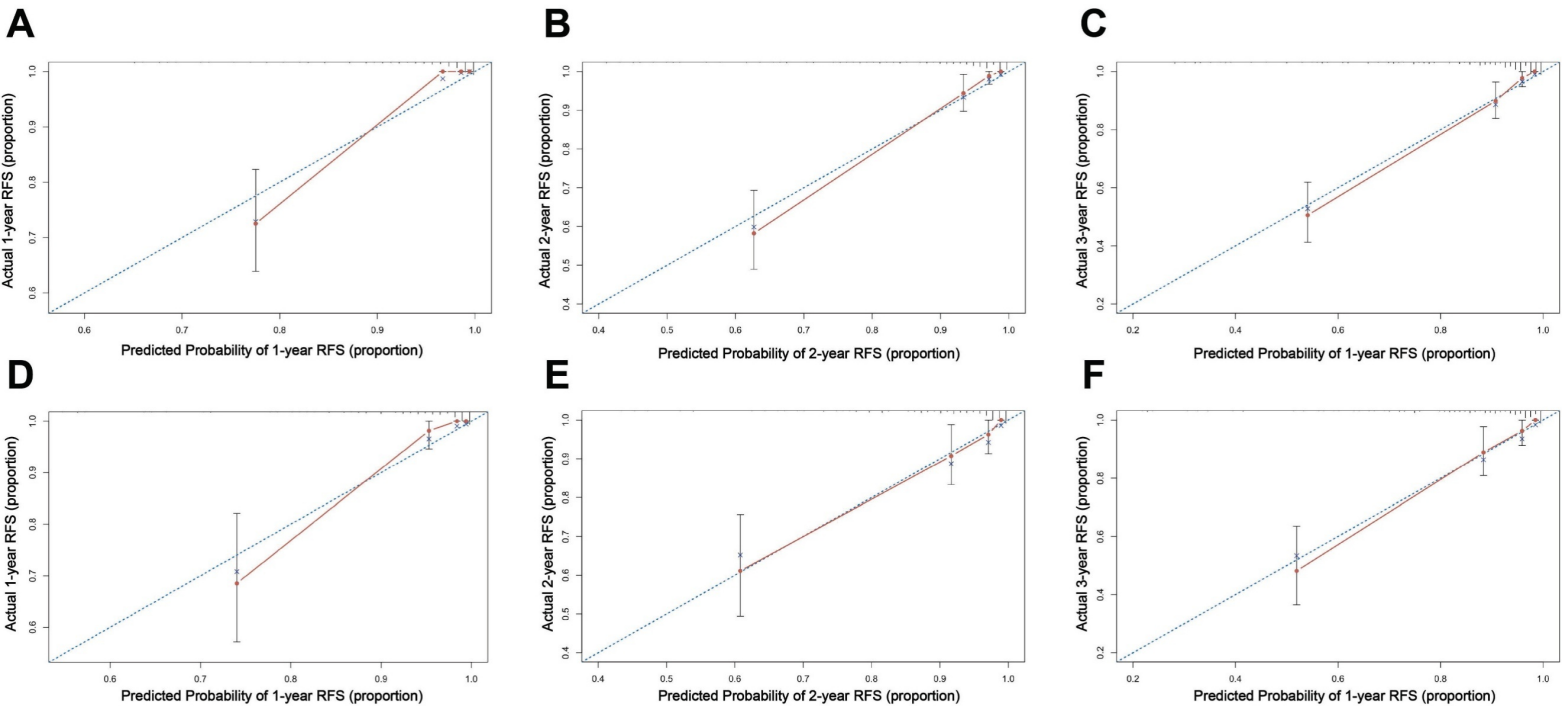
Calibration curve of the nomogram model for predicting 1-, 2-, and 3-year RFS in the training set (A-C) and external validation set (D-F).

The established nomogram model was further verified using C-index and AUC values (Figure 3B, C). The nomogram for predicting RFS in the training cohort had a C-index of 0.875 (95% CI 0.836-0.914), and the C-index remained stable in the external validation cohort [C-index of 0.859 (95% CI 0.806-0.912)]. The calibration curves in the training and validation cohorts closely aligned with the ideal line, showing adequate agreement between the predictive nomogram and actual observations (Figure 4).

### Comprehensive evaluation of a novel nomogram model for predicting RFS in endometrial cancer

**Figure 5:**
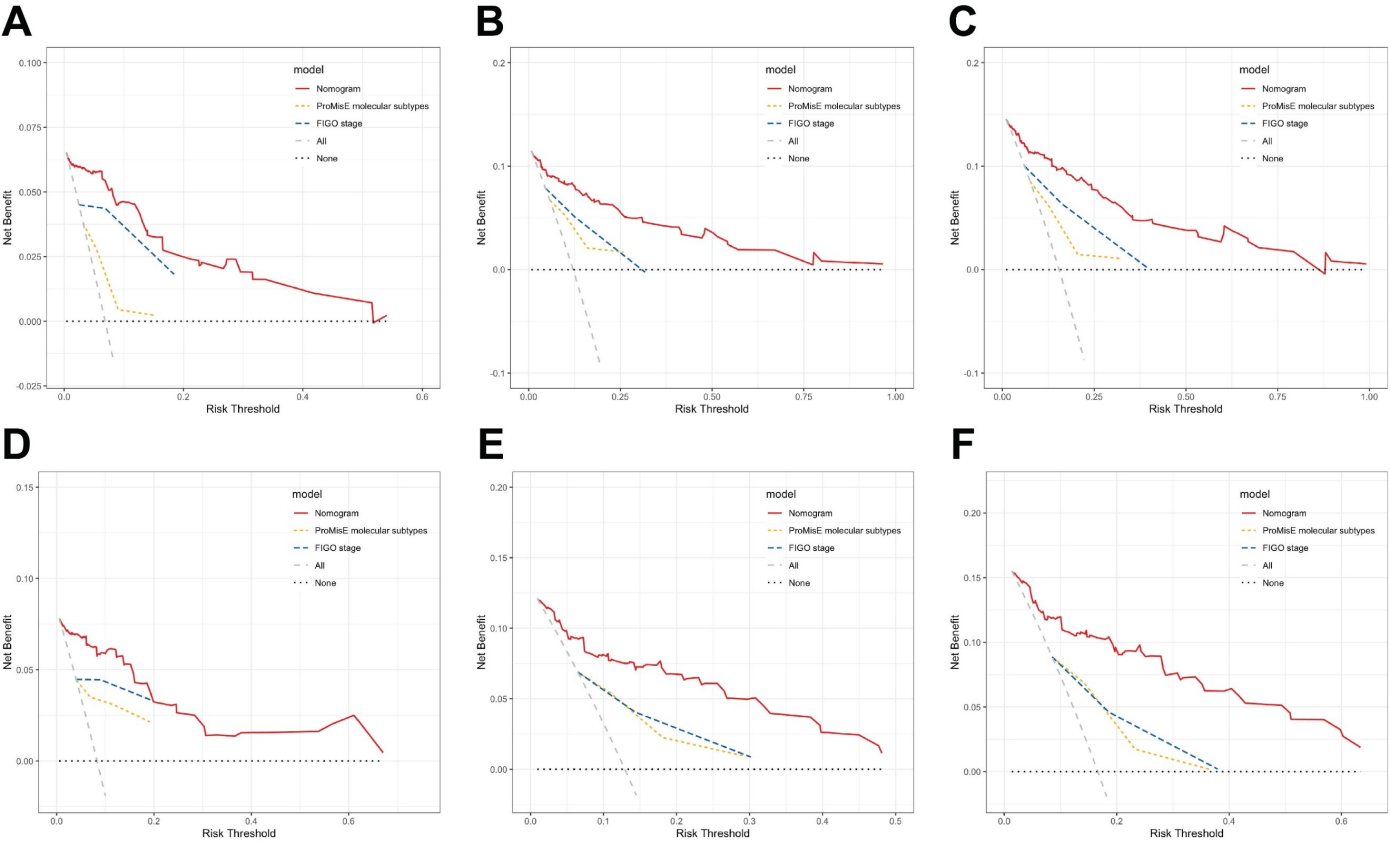
DCA curves of various risk stratification systems including the FIGO staging system, ProMisE molecular classification, and the established nomogram, for the prediction of 1-, 2-, and 3-year RFS in the training (A-C) and validation sets (D-F).

According to DCA curves, the value of the established model in clinical practice has been thoroughly evaluated over time (Figure 5). The results indicate that in both the training and validation cohorts, the threshold probabilities for the standardized net benefit of the predictive model are significantly higher than those of individual variables (such as FIGO stage or ProMisE molecular classification).

Additionally, the differences in C-index were calculated to compare the accuracy of the nomogram with the FIGO criteria-based tumor staging and ProMisE molecular classifier. These results indicate that the novel nomogram demonstrates a higher prognostic power for RFS in endometrial cancer compared to the FIGO staging system and the ProMisE molecular classification (Table S3). Furthermore, when comparing the nomogram model with other risk stratification systems, the results show that our model provides more accurate predictions for RFS in endometrial cancer (Table 4).

### Survival analysis

**Figure 6:**
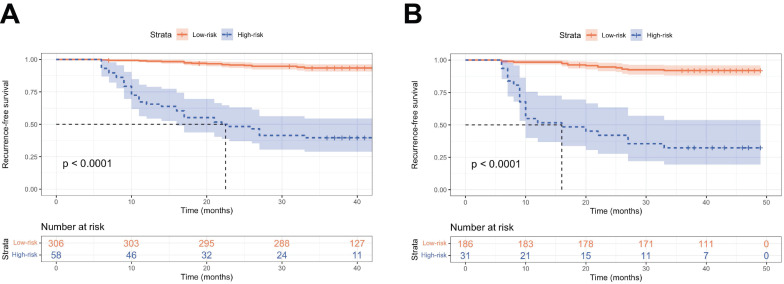
Kaplan-Meier curves of the low- and high-risk groups stratified by the predictive model. RFS curves in the training set (A) and the validation set (B).

To further validate the performance of the nomogram model in stratifying risk, patients from two cohorts were stratified into low-risk (total score ≤ 295.6 pts) and high-risk (total score > 295.6 pts) groups using the cutoff value of nomogram-generated scores for RFS (Figure S3). Kaplan-Meier curves demonstrated that patients classified as the high-risk had significantly worse RFS compared to those in the low-risk group (*P* < 0.001, Figure 6).

## Discussion

Over the last couple of decades, the contributions of the immune system and inflammation to the development, progression, and treatment of cancer have garnered enormous attention and interest 24. HALP, as a personalized oncology approach reflecting the overall nutrition-immune-inflammatory status, can be utilized to predict the prognosis of cancer patients and offer valuable insights for treatment decisions. In this study, we included a total of 528 patients with endometrial cancer from four large medical institutions in southern and central-western China to investigate the prognostic significance of the pretreatment HALP score for survival outcomes. Our findings indicated that a low pretreatment HALP score serves as an unfavorable prognostic biomarker for cancer recurrence. Additionally, multiple clinicopathological data, preoperative blood parameters, and molecular classifications of endometrial cancer were obtained. The Cox proportional hazards method identified age, FIGO stage, tumor grade, LVSI, Ca125, ProMisE molecular classification, HALP score, and adjuvant treatment as independent prognostic factors for endometrial cancer recurrence. Furthermore, we developed a novel nomogram model to predict the RFS of operable endometrial cancer patients.

HALP integrates routine hematological parameters, including hemoglobin, albumin, lymphocytes, and platelets to effectively reflect the individual's inflammation, nutrition, and immune status. It has been confirmed in clinical practice to predict the prognosis of ovarian cancer 25, hepatocellular carcinoma 25, 26, breast cancer 19, and esophageal cancer 27, etc. However, the mechanisms underlying HALP have not been fully elucidated, and the physiological and pathological roles of these peripheral blood parameters may explain this to some extent. Hemoglobin serves as a crucial indicator of anemia, which is particularly common in cancer patients, especially in the advanced stages of the disease. Currently, it is believed that tumor cells produce a significant amount of pro-inflammatory cytokines, including IL-1β and IL-6, as they adapt to the surrounding environment 28. These cytokines not only intensify the inflammatory response but also disrupt the cell's iron utilization, resulting in reduced erythropoietin production and decreased sensitivity of erythroid precursors to erythropoietin, ultimately affecting the generation and maturation of red blood cells 29. The decrease in hemoglobin levels leads to anemia, resulting in tumor hypoxia and treatment resistance, which are closely associated with a worse prognosis in cancer patients 30. An increasing amount of evidence highlights the link between nutritional status and malignant tumors 31. Serum albumin, as an index for assessing nutritional status, is widely used in clinical practice due to its cost-effectiveness, non-invasiveness, and easy accessibility. Given that cancer is inherently a chronic wasting disease, patients frequently encounter malnutrition and hypoproteinemia 32. Multiple studies have indicated that severe hypoproteinemia can lead to a poor prognosis in cancer patients 33, 34. Elevated levels of lymphocytes in the peripheral blood directly reflect host anti-tumor immunity. Lymphocytes are the primary executors of host anti-tumor defense and immune surveillance. CD8+ T cells, as the primary effector cells in anti-tumor immune responses, have the ability to directly eliminate tumor cells through signaling pathways involving Fas and Fas ligand (FasL). Furthermore, these immune cells can secrete cytokines like interferon-gamma (IFN-γ) and tumor necrosis factor-alpha (TNF-α), indirectly causing cell death or apoptosis, thereby improving patient prognosis 35. Research has confirmed that there is an interaction between platelets and tumor cells, which can promote tumor growth by enhancing angiogenesis and inducing the proliferation of stromal cells. Additionally, platelets regulate the inflammatory response and alter the tumor microenvironment through both anti-inflammatory and pro-inflammatory mechanisms 36. Pro-tumorigenic inflammation can reshape the tumor microenvironment towards a state that supports tumor growth and invasion by blocking anti-tumor immunity, and promoting cancer progression by exerting cytokines and signals on surrounding epithelial and stromal cells 37. Some tumor cells, while not characterized by significant T cell infiltration or functional activation, may still exhibit pro-tumorigenic properties by upregulating inflammatory mediators and recruiting other immune cells such as macrophages, monocytes, neutrophils, etc. A substantial body of epidemiological studies also underscores the critical role of inflammation in promoting tumor initiation, growth, and progression 38. Collectively, the HALP score can serve as a novel prognostic factor to assist in evaluating cancer patient prognosis 39.

In recent years, TCGA molecular subtyping has been shown to significant value in the prognostic evaluation of endometrial cancer and has been incorporated into the lastest guidelines. However, due to the uneven development of regional healthcare, not all laboratories are currently able to perform NGS testing (TCGA molecular classification) for all endometrial cancer cases. During this transition phase, in situations with limited resources, priority may be given to immunohistochemical staining for at least 2 (PMS2 and MSH6) or preferably 4 (PMS2, MLH1, MSH6 and MSH2) of the MMR proteins, with immunostaining of p53 serving as a good surrogate for MSI-H and *TP53* mutation. Recently, scholars have proposed and confirmed the effectiveness of the ProMisE molecular classification system as an alternative method to NGS testing. Research has confirmed that the ProMisE classification system significantly reduces testing costs and technical barriers by detecting the exonuclease domain of *POLE* in exons 9-14, and IHC for MMR and p53 proteins. It has demonstrated a high level of consistency between diagnostic endometrial samples (biopsy or curettage) and whole uterine specimens. Consistent with a previous study of 452 women with endometrial cancer 10, we found that the ProMisE molecular classification was independently associated with RFS.

Accumulating evidence indicates that the prognosis and progression of endometrial cancer are associated not only with traditional clinicopathological variables but also with systemic inflammatory responses. Recent studies have reported a connection between cancer recurrence and the HALP score in stage I-III endometrial cancer. In recent years, there has been considerable interest in developing predictive models that utilize multiple markers rather than relying on single inflammatory markers. This retrospective study investigated the clinical significance of the HALP score in stage I-III endometrial cancer. By integrating traditional clinicopathological parameters, the HALP score, and ProMisE molecular subtyping, we constructed a nomogram model for a comprehensive evaluation of patient prognosis. The model's performance was further evaluated and externally validated using Harrell's C-index, AUC value, calibration curve, and DCA curve. Additionally, the completeness of patient information in the dataset and the model's construction based on extensive data from multiple national medical institutions further enhanced the reliability and universality of the model. Furthermore, when compared with existing risk stratification systems, our model demonstrates more accurate predictions for RFS in endometrial cancer patients.

However, there remained some limitations in our study. Firstly, due to the retrospective nature of this study, many endometrial cancer patients in early clinical stages and low-risk categories were not recommended for genetic testing or *POLE* hotspot testing. This resulted in the loss of information regarding *POLE* variants and their exclusion from the study. Consequently, this may have led to an overall advanced FIGO stage and an increased prevalence of high-risk factors in the patient cohort of the present study. Moreover, the retrospective design introduces potential biases. Secondly, the sample size was relatively small, highlighting the need for future multi-center studies with expanded sample sizes. Additionally, given the significant differences in genetic backgrounds and lifestyles between Eastern and Western patients, this model may be more applicable to Eastern patients. Therefore, future research should further validate its applicability and predictive performance across diverse populations through prospective international multicenter clinical trials.

## Conclusion

Our study demonstrates that HALP is an independent prognostic factor in endometrial cancer, which provides a nutrition-immune-inflammation perspective to understand the recurrence of the disease. Additionally, we constructed a nomogram for RFS by integrating traditional clinicopathological parameters, HALP score, and ProMisE molecular classification. This model will assist clinicians in identifying high-risk patients and establishing individualized treatments.

## Supplementary Material

Supplementary figures and tables.

## Figures and Tables

**Table 1:** Demographic, clinicopathologic, and treatment variables in patients with stage I-III endometrial cancer

Admission variables	Total (n = 582)	Training cohort (n = 365)	External validation cohort (n = 217)	*P* value
**Age, years**	54.26 ± 9.719	54.20 ± 9.781	54.36 ± 9.637	0.844
**BMI, kg/m^2^**	24.10 ± 3.515	24.12 ± 3.594	24.07 ± 3.387	0.887
**FIGO stage**				0.936
I	367 (63.0)	230 (63.0)	137 (63.1)	
II	72 (12.4)	44 (12.1)	28 (12.9)	
III	143 (24.6)	91 (24.9)	52 (24.0)	
**Present of LVSI**				0.462
Negative	389 (66.8)	248 (67.9)	141 (65.0)	
Positive	193 (33.2)	117 (32.1)	76 (35.0)	
**Myometrial invasion**				0.589
< 50%	372 (63.9)	229 (62.7)	143 (65.9)	
≥ 50%	210 (36.1)	136 (37.3)	74 (34.1)	
**Tumor grade**				0.430
G1-G2	451 (77.5)	279 (76.4)	172 (79.3)	
G3	131 (22.5)	86 (23.6)	45 (20.7)	
**Histology**				0.776
Endometrioid	494 (84.9)	311 (85.2)	183 (84.3)	
Non-Endometrioid	88 (15.1)	54 (14.8)	34 (15.7)	
**Serum Ca125**				0.740
≤ 35 U/ml	431 (74.1)	272 (74.5)	159 (73.3)	
> 35 U/ml	151 (25.9)	93 (25.5)	58 (26.7)	
**ProMisE molecular**				0.600
p53wt	257 (44.2)	155 (42.5)	102 (47.0)	
MMR-D	148 (25.4)	94 (25.8)	54 (24.9)	
*POLE* EDM	59 (10.1)	41 (11.2)	18 (8.3)	
p53-abn	118 (20.3)	75 (20.5)	43 (19.8)	
**HALP score**				0.510
High-HALP	364 (62.5)	232 (63.6)	132 (60.8)	
Low-HALP	218 (37.5)	133 (36.4)	85 (39.2)	
**Adjuvant treatment**				0.325
Follow-up	193 (33.2)	122 (33.4)	71 (32.7)	
Radiotherapy only	169 (29.0)	111 (30.4)	58 (26.7)	
Chemotherapy only	20 (3.4)	9 (2.5)	11 (5.1)	
Radio-chemotherapy	200 (34.4)	123 (33.7)	77 (35.5)	
**Recurrence**				0.690
No	490 (84.2)	309 (84.7)	181 (83.4)	
Yes	92 (15.8)	56 (15.3)	36 (16.6)	
**RFS time, month**	36.22 ± 9.964	35.58 ± 9.266	37.29 ± 10.979	0.056

**Abbreviations:** LVSI, lymphovascular space invasion; G1-G2, grade 1/2; G3, grade 3; HALP, hemoglobin-albumin-lymphocyte-platelet; *POLE* EDM, *POLE* exonuclease domain mutation; p53-wt, p53 wide-type; p53-abn, p53-abnormal; MMR-D, identifying defective expression of any mismatch repair protein by IHC (i.e. dMMR).

**Table 2:** Comparison of clinicopathological parameters between the high-HALP group and the low-HALP group (n = 582)

Admission variables	Training cohort		*P* value	Validation cohort		*P* value
	Low-HALP	High-HALP		Low-HALP	High-HALP	
**Age, years**	54.21 ± 10.574	54.24 ± 9.923	0.979	53.04 ± 8.930	55.22 ± 10.006	0.103
**BMI, kg/m^2^**	23.53 ± 3.48	24.46 ± 3.62	0.017	23.51 ± 3.059	24.44 ± 3.536	0.048
**FIGO stage**			0.016			0.049
I	72 (54.1)	158 (68.1)		47 (54.6)	90 (68.7)	
II	17 (12.8)	27 (11.6)		11 (12.8)	17 (13.0)	
III	44 (33.1)	47 (20.3)		28 (32.6)	24 (18.3)	
**Present of LVSI**			0.211			0.069
Negative	85 (63.9)	163 (70.3)		49 (57.6)	92 (69.7)	
Positive	48 (36.1)	69 (29.7)		36 (42.4)	40 (30.3)	
**Myometrial invasion**			<0.001			< 0.001
< 50%	68 (51.1)	161 (69.4)		45 (52.9)	98 (74.2)	
≥ 50%	65 (48.9)	71 (30.6)		40 (47.1)	34 (25.8)	
**Tumor grade**			0.670			0.638
G1-G2	100 (75.2)	179 (77.2)		66 (77.6)	106 (80.3)	
G3	33 (24.8)	53 (22.8)		19 (22.4)	26 (19.7)	
**Histology**			0.836			0.794
Endometrioid	114 (85.7)	197 (84.9)		71 (83.5)	112 (84.8)	
Non-Endometrioid	19 (14.3)	35 (15.1)		14 (16.5)	20 (15.2)	
**Serum Ca125**			<0.001			0.097
≤ 35 U/ml	86 (64.7)	186 (80.2)		57 (67.1)	102 (77.3)	
> 35 U/ml	47 (35.3)	46 (19.8)		28 (32.9)	30 (22.7)	
**ProMisE molecular**			0.282			0.412
p53wt	53 (39.8)	102 (44.0)		35 (41.2)	67 (50.8)	
MMR-D	34 (25.6)	60 (25.9)		24 (28.2)	30 (22.7)	
*POLE* EDM	12 (9.0)	29 (12.4)		6 (7.1)	12 (9.1)	
p53-abn	34 (25.6)	41 (17.7)		20 (23.5)	23 (17.4)	
**Adjuvant treatment**			0.885			0.493
Follow-up	38 (28.6)	84 (36.2)		23 (27.1)	48 (36.4)	
Radiotherapy only	43 (32.3)	68 (29.3)		26 (30.6)	32 (24.2)	
Chemotherapy only	3 (2.3)	6 (2.6)		4 (4.7)	7 (5.3)	
Radio-chemotherapy	49 (36.8)	74 (31.9)		32 (37.6)	45 (34.1)	

**Abbreviations:** FIGO, International Federation of Gynecology and Obstetrics; LVSI, lymphatic vessel space invasion; G1-G2, grade 1/2; G3, grade 3; *POLE* EDM, polymerase epsilon (*POLE*) exonuclease domain mutation; p53-abn, p53-abnormal; MMR-D, deficient mismatch repair; HALP, hemoglobin, albumin, lymphocyte, and platelet.

**Table 3:** Factors associated with RFS in univariable and multivariable analysis

Variable	Univariable analysis		Multivariable analysis	
	HR (95% CI)	*P* value	HR (95% CI)	*P* value
**Age at diagnosis** (≥ 60years vs < 60years)	2.022 (1.170-3.494)	0.012	1.820 (1.005-3.293)	0.048
**FIGO stage**		< 0.001		0.113
Ι	Reference	-	Reference	-
ΙΙ	1.789 (0.650-4.921)	0.260	1.298 (0.451-3.734)	0.629
ΙΙΙ	7.697 (4.211-14.070)	< 0.001	2.309 (1.033-5.164)	0.042
**Tumor grade** (G3 vs G1-G2)	7.379 (4.267-12.760)	< 0.001	2.329 (1.082-5.016)	0.031
**Histology** (Non-Endometrioid vs Endometrioid)	4.604 (2.690-7.879)	< 0.001	1.073 (0.542-2.124)	0.841
**LVSI** (Positive vs Negative)	7.605 (4.151-13.935)	< 0.001	2.304 (1.022-5.195)	0.044
**Myometrial invasion** (≥ 50% vs <50%)	4.085 (2.331-7.159)	< 0.001	1.716 (0.924-3.186)	0.087
**Serum Ca125** (> 35U/ml vs ≤ 35U/ml)	3.942 (2.330-6.667)	< 0.001	2.100 (1.135-3.885)	0.018
**ProMisE molecular subgroup**		< 0.001		0.067
p53wt	Reference	-	Reference	-
MMR-D	3.172 (1.464-6.871)	0.003	1.520 (0.673-3.434)	0.314
*POLE* EDM	0.370 (0.047-2.887)	0.343	0.388 (0.047-3.221)	0.381
p53abn	6.645 (3.214-13.736)	< 0.001	2.551 (1.093-5.954)	0.030
**HALP score** (Low HALP vs High HALP)	3.018 (1.765-5.161)	< 0.001	2.099 (1.182-3.728)	0.011
**Adjuvant treatment** (No vs Yes)	1.579 (0.863-2.892)	0.006	0.352 (0.160-0.774)	0.009

**Abbreviations:** HR, hazard ratio; CI, confidence interval; LVSI, lymphovascular space invasion; G1-G2, grade 1/2; G3, grade 3; *POLE* EDM, polymerase epsilon (*POLE*) exonuclease domain mutation; p53-wt, p53 wide-type; p53-abn, p53-abnormal; MMR-D, deficient mismatch repair; HALP, hemoglobin-albumin-lymphocyte-platelet.

**Table 4:** The predictive performance of diverse risk stratification models for predicting endometrial cancer recurrence

Risk stratification	Key predictors of the prediction model	C-index (95% CI)	
		Training set	Validation set
Model A 40	A nomogram including age, surgical staging, histological grade, LVSI, FIGO staging.	0.801 (0.740-0.862)	0.786 (0.725-0.847)
Model B 41	A risk stratification system based on systemic immune-inflammation index (SII).	0.638 (0.575-0.701)	0.602 (0.526-0.678)
Model C 42	A nomogram model including age, Ca125, FIGO staging, LVSI, and P53.	0.810 (0.753-0.867)	0.796 (0.731-0.861)
Our model	A nomogram including age, FIGO staging, tumor grade, LVSI, serum Ca125, ProMisE molecular subgroup, HALP score, and adjuvant treatment.	0.875 (0.836-0.914)	0.859 (0.806-0.912)

**Abbreviation:** FIGO, International Federation of Gynecology and Obstetrics; LVSI, lymphatic vessel space invasion; SII, systemic immune-inflammation index; HALP, hemoglobin, albumin, lymphocyte, and platelet. **Note:** Comparing predictive performance among different models using the C-index, SII = platelet counts × neutrophil counts/lymphocyte counts.
