# Supplementary material for: Development and validation of a prediction model for recurrence based on ProMisE molecular classifier and immune-inflammatory- nutritional score in endometrial carcinoma: a retrospective multiple-center study
Source: Int J Med Sci. 2025 Aug 16;22(14):3789–801. doi: 10.7150/ijms.107134 (PMC12434823; doi:10.7150/ijms.107134)
Supplement: Supplementary file 1 — Supplementary figures and tables. [file ijmsv22p3789s1.pdf]

Supplementary Information

Table S1. General characteristics of haematological parameters and inflammatory indices in patients with endometrial cancer (n = 528)

Characteristic	Whole population	Training cohort	Validation cohort	*P* value
**HGB (g/L)**				0.637
Mean ±(SD)	119.90 ± (16.54)	120.15 ± (16.42)	119.47 ± (16.76)	
Median (IQR)	123.00 (111.00-131.00)	123.00 (111.00-131.00)	123.00 (112.00-131.00)	
**Albumin (g/L)**				0.527
Mean ±(SD)	41.62 ± (5.10)	41.52 ± (4.93)	41.80 ± (5.37)	
Median (IQR)	42.00(39.00-45.00)	42.00 (39.00-45.00)	43.00 (39.00-45.00)	
**Lymphocyte (x10^9/L)**				0.808
Mean ±(SD)	1.68 ± (0.57)	1.69 ± (0.58)	1.65 ± (0.55)	
Median (IQR)	1.66 (1.25-2.04)	1.68 (1.25-2.09)	1.61 (1.25-1.99)	
**Blood platelet (x10^9/L)**				0.266
Mean ±(SD)	221.59 ± (75.12)	218.91 ± (75.32)	226.08 ± (74.73)	
Median (IQR)	211.50 (176.00-259.00)	206.00 (175.5-249.00)	214 (177.00-262.50)	
**HALP score**				0.344
Mean ±(SD)	42.67 ± (23.51)	43.38 ± (23.66)	41.47 ± (23.27)	
Median (IQR)	41.58 (24.30-53.33)	42.39 (25.86-53.51)	40.39 (23.70-52.58)	

Abbreviations: HGB, Hemoglobin; HALP, hemoglobin, albumin, lymphocyte, and platelet. IQR, interquartile range.

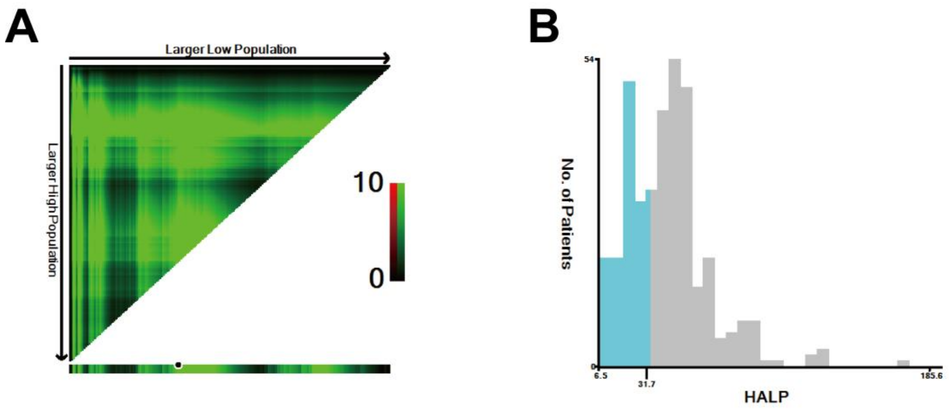

Figure S1. The optimal cutoff value of HALP score determined by X-tile (value = 31.70).

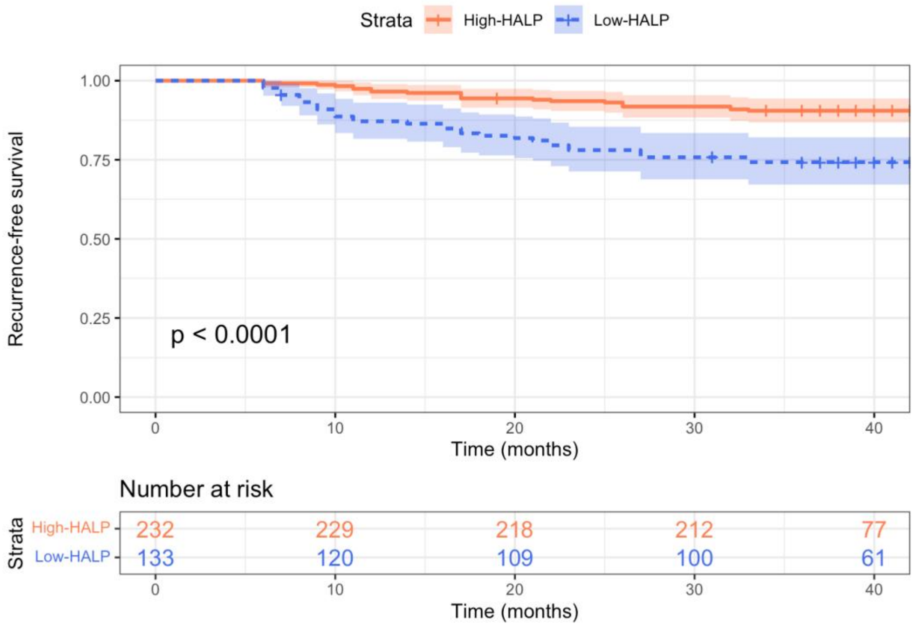

Figure S2. RFS of stage I-III endometrial cancer stratified by HALP score.

Table S2. Variable risk score and RFS probability prediction in patients with stage I-III endometrial cancer

Characteristics	Risk score	Total score	Probability of RFS
**Age**		**Total Points for 1-year RFS**	**Probability of 1-year RFS**
< 60 years	0	357	0.2
≥ 60 years	33	342	0.3
**FIGO staging**		328	0.4
I	0	314	0.5
II	15	299	0.6
III	42	281	0.7
**Grade**		258	0.8
G1-G2	0	220	0.9
G3	49	**Total Points for 2-year RFS**	**Probability of 2-year RFS**
**LVSI**		321	0.2
Negative	0	306	0.3
Positive	49	292	0.4
**Ca125**		278	0.5
≤ 35 U/ml	0	263	0.6
> 35 U/ml	36	245	0.7
**ProMisE molecular subgroup**		222	0.8
p53-wt	51	184	0.9
POLE EDM	0	**Total Points for 3-year RFS**	**Probability of 3-year RFS**
MMR-D	75	303	0.2
p53-abn	100	289	0.3
**Adjuvant treatment**		275	0.4
Yes	0	261	0.5
No	50	246	0.6
**HALP score**		228	0.7
High	0	204	0.8
Low	39	167	0.9

Abbreviations: FIGO, International Federation of Gynecology and Obstetrics; LVSI, lymphovascular space invasion; G1-G2, grade 1/2; G3, grade 3; *POLE* EDM, polymerase epsilon (*POLE*) exonuclease domain mutation; p53-wt, p53 wide-type; p53-abn, p53-abnormal; MMR-D, deficient mismatch repair; HALP, hemoglobin-albumin-lymphocyte-platelet.

Table S3. C-index for the nomogram and model variables in the training and external validation cohorts

Risk stratification system	Training cohort		External validation cohort	
	C-index	95% CI	C-index	95% CI
**ProMisE molecular subgroup**	0.678	(0.611-0.745)	0.691	(0.607-0.775)
**FIGO stage[a]**	0.736	(0.673-0.799)	0.688	(0.604-0.772)
**Nomogram Model**	0.875	(0.836-0.914)	0.859	(0.806-0.912)

[a] Adopt FIGO 2023 staging criteria for endometrial cancer.

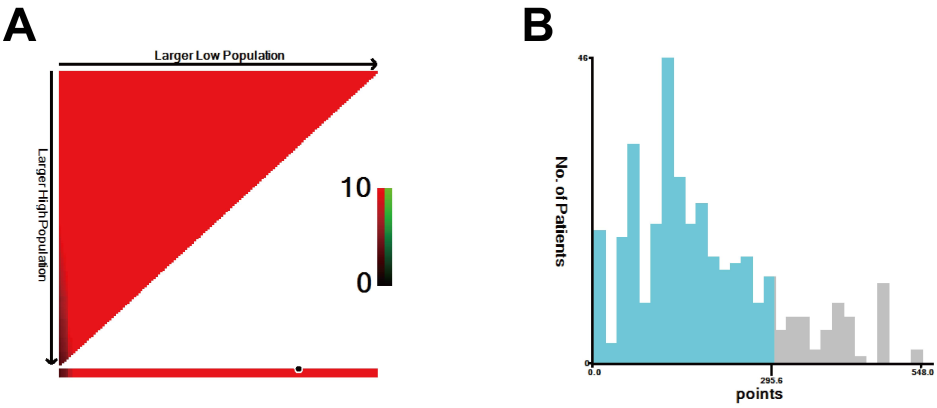

Figure S3. The optimal cutoff value for nomogram risk score determined by X-tile; and the RFS of endometrial cancer patients stratified by the scores (optimal cutoff value = 295.6 pts).

Supplementary materials

Description: Related Computerized Programs for Nomogram Development Using R

APPENDIX

R code for developing the RFS nomogram model and calculating the nomogram score for each patient

```
library(survival)
library(rms)
dd <- datadist(data)
options(datadist = "dd")
F <- cph(Surv(time, status) ~ Age + FIGO + Grade + LVSI + Ca125 +
ProMisE_molecular +HALP + Adjuvant_treatment, data = data, x = TRUE, y =
TRUE, surv = TRUE)
# Developing nomogram models
survival <- Survival(f)
survival1 <- function(x)survival(12,x)
survival2 <- function(x)survival(36,x)
survival3 <- function(x)survival(60,x)
nom <- nomogram(f, fun=list(survival1,survival2,survival3), lp = FALSE, fun.at =
c(0.1, seq(0.1,0.9,by=0.1), 0.9), funlabel = c("1-year RFS", "3-year RFS","5-year
RFS"))
plot(nom)
# Calculating the nomogram score for each patient
library(nomogramFormula)
data = na.omit(data)
dd<-datadist(data)
options(datadist="dd")
f <- cph(Surv(time, status) ~ Age + FIGO + Grade + LVSI + Ca125 +
ProMisE_molecular +HALP + Adjuvant_treatment, data=data, x=TRUE, y=TRUE,
surv = TRUE)
```

```
results <- formula_rd(nom)
results$formular
data$points <- points_cal(formula = results$formula, rd=data)
```

For calculating the C-index and 95% *CI* in both the training and validation cohorts

```
library(survival)
library(rms)
fit<-coxph(Surv(time, status) ~ Age + FIGO + Grade + LVSI + Ca125 +
ProMisE_molecular +HALP + Adjuvant_treatment, data = Ytraindata)
survConcordance(Surv(Ytraindata$time,Ytraindata$status)~predict(fit,Ytraindata))
survConcordance(Surv(Ytestdata$time,Ytestdata$status)~predict(fit,Ytestdata))
# Other coding methods
library(rms)
library(foreign)
library(survival)
ddist <- datadist(data)
options(datadist = 'ddist')
# C-index confidence interval: C-index-1.96*se/C-index+1.96*se
fmla1 <- as.formula(Surv(time, status) ~ Age + FIGO + Grade + LVSI + Ca125 +
ProMisE_molecular +HALP + Adjuvant_treatment)
cox2 <- coxph(fmla1, data=data)
summary(cox2)
```

R code for ROC curve for the nomogram model

```
cox_m <- coxph(Surv(time, status) ~ Age + FIGO + Grade + LVSI + Ca125 +
ProMisE_molecular +HALP + Adjuvant_treatment, data = data)
cox_m1<-step(cox_m, direction = "both")
risk_score<-predict(cox_m1, type="risk", newdata=data)
risk_level<-as.vector(ifelse(risk_score > median(risk_score), "High", "Low"))
#colnames(data) <- c("id", "survival_time", "status", "risk_score", "predict_time")
```

```
write.table(cbind(id=rownames(cbind(data$time, data$status, risk_score, risk_level)),
cbind(data$time,data$status,risk_score,risk_level)),"risk_score.txt",sep="\t",quote=F,r
ow.names=F)
# Plotting the ROC curves for 1-year RFS
library(survivalROC)
colnames(data) <- c("id", "survival_time", "status", "risk_score", "predict_time")
predict_time <- 12*1
myroc <- survivalROC(Stime = data$survival_time, status=data$status, marker =
data$risk_score, predict.time = predict_time, method="KM")
pdf("ROC_1-year_RFS_in_Training_set.pdf")
plot(myroc$FP, myroc$TP, type="l", xlim=c(0,1), ylim=c(0,1), col="blue",
        xlab="FP", ylab="TP", lwd=2.5, main=paste("1-year
RFS","AUC=",round(myroc$AUC,3)))
abline(0,1)
dev.off()
# Plotting the ROC curves for 3-year RFS
library(survivalROC)
colnames(data) <- c("id", "survival_time", "status", "risk_score", "predict_time")
predict_time <- 12*3
myroc <- survivalROC(Stime = data$survival_time, status=data$status, marker =
data$risk_score, predict.time = predict_time, method="KM")
pdf("ROC_3-year_RFS_in_Training_set.pdf")
plot(myroc$FP, myroc$TP, type="l", xlim=c(0,1), ylim=c(0,1), col="blue",
        xlab="FP", ylab="TP", lwd=2.5, main=paste("3-year
RFS","AUC=",round(myroc$AUC,3)))
abline(0,1)
dev.off()
# Plotting the ROC curves for 5-year RFS
predict_time<-12*5
```

```
myroc<-survivalROC(Stime = data$survival_time, status = data$status, marker =
data$risk_score, predict.time=predict_time,method="KM")
pdf("ROC_5-year RFS in Training set.pdf")
plot(myroc$FP, myroc$TP, type="l", xlim=c(0,1), ylim=c(0,1), col="red",
     xlab="FP", ylab="TP", lwd=2.5, main=paste("5-year RFS","AUC=",
round(myroc$AUC,3)))
abline(0,1)
dev.off()
```

R code for calibration curve of the nomogram model

```
library(survival)
library(rms)
Ytraindata <- data
f1 <- cph(Surv(Ytraindata$time, Ytraindata$status==1)~Age + FIGO + Grade + LVSI
+ Ca125 + ProMisE_molecular +HALP + Adjuvant_treatment, data = Ytraindata,
x=TRUE, y=TRUE, surv=TRUE, time.inc=3*12)
cal<-calibrate(f1, cmethod = 'KM', method = "boot", u=3*12, m=120, B=1000)
plot(cal, #Input the calibrate model
     lwd=1, #Line segment width
     lty=1, #Line segment type
     errbar.col=c(rgb(0,0,0,maxColorValue = 255)), #Specify the error line color
     xlim = c(0.7,1),ylim = c(0.7,1), #Set the axis range
     xlab ="Nomogram-Predicted Probability of 3-year RFS (proportion)", #Set the
x-axis label
     ylab="Actual 3-year RFS (proportion)", #Set the y-axis label
     col=c(rgb(255,0,0,maxColorValue =255))) abline(0,1,lty = 3, lwd = 2, col =
c(rgb(0,118,192,maxColorValue=255)))
lines(cal[,c('mean.predicted','KM')], type = 'b',lwd = 2, col =
c(rgb(192,98,83,maxColorValue = 255)), pch = 16)
abline(0,1,lty = 3,lwd = 2,col = c(rgb(0,118,192,maxColorValue=255)))
```

```
lines(cal[,c('mean.predicted','KM')], type = 'b',lwd = 2, col =
c(rgb(192,98,83,maxColorValue = 255)),pch = 16)
#1_year
f1 <- cph(Surv(Ytraindata$time, Ytraindata$status==1)~Age + FIGO + Grade + LVSI
+ Ca125 + ProMisE_molecular +HALP + Adjuvant_treatment, data = Ytraindata,
x=TRUE, y=TRUE, surv=TRUE, time.inc=1*12)
cal<-calibrate(f1, cmethod = 'KM', method = "boot", u=1*12, m=120, B=1000)
plot(cal, lwd=1, lty=1, errbar.col=c(rgb(0,0,0,maxColorValue = 255)), xlim = c(0.8,1),
ylim = c(0.7,1), xlab ="Nomogram-Predicted Probability of 1-year RFS (proportion)",
ylab="Actual 1-year RFS (proportion)", col=c(rgb(255,0,0, maxColorValue =255)))
abline(0,1,lty = 3, lwd = 2, col = c(rgb(0,118,192,maxColorValue=255)))
lines(cal[,c('mean.predicted','KM')], type = 'b',lwd = 2, col =
c(rgb(192,98,83,maxColorValue = 255)), pch = 16)
#5_year
f1 <- cph(Surv(Ytraindata$time, Ytraindata$status==1)~Age + FIGO + Grade + LVSI
+ Ca125 + ProMisE_molecular +HALP + Adjuvant_treatment, data = Ytraindata,
x=TRUE, y=TRUE, surv=TRUE, time.inc=5*12)
cal<-calibrate(f1, cmethod = 'KM', method = "boot", u=5*12, m=120, B=1000)
plot(cal, lwd=1, lty=1, errbar.col=c(rgb(0,0,0,maxColorValue = 255)), xlim = c(0.8,1),
ylim = c(0.7,1), xlab ="Nomogram-Predicted Probability of 1-year RFS (proportion)",
ylab="Actual 1-year RFS (proportion)", col=c(rgb(255,0,0, maxColorValue =255)))
abline(0,1,lty = 3, lwd = 2, col = c(rgb(0,118,192,maxColorValue=255)))
lines(cal[,c('mean.predicted','KM')], type = 'b',lwd = 2, col =
c(rgb(192,98,83,maxColorValue = 255)), pch = 16)
```

For calculating the C-index and 95% *CI* of model variables for RFS

```
# ProMisE molecular subgroup
library(survival)
library(rms)
fit1 <- coxph(Surv(time,status)~ProMisE_molecular, data = Ytraindata)
```

```
survConcordance(Surv(Ytraindata$time,Ytraindata$status)~predict(fit1, Ytraindata))
survConcordance(Surv(Ytestdata$time,Ytestdata$status)~predict(fit1, Ytestdata))

# FIGO stage
library(survival)
library(rms)
fit1< - coxph(Surv(time,status)~FIGO_stage, data = Ytraindata)
survConcordance(Surv(Ytraindata$time,Ytraindata$status)~predict(fit1, Ytraindata))
survConcordance(Surv(Ytestdata$time,Ytestdata$status)~predict(fit1, Ytestdata))
```

For calculating the C-index and 95% *CI* of different risk stratification systems for RFS

```
# Model A
library(survival)
library(rms)
fit1 <- coxph(Surv(time,status)~age + surgical_staging + Grade + LVSI + FIGO, data = Ytraindata)
survConcordance(Surv(Ytraindata$time,Ytraindata$status)~predict(fit1, Ytraindata))
survConcordance(Surv(Ytestdata$time,Ytestdata$status)~predict(fit1, Ytestdata))

# Model B
library(survival)
library(rms)
fit1 <- coxph(Surv(time,status)~SII, data = Ytraindata)
survConcordance(Surv(Ytraindata$time,Ytraindata$status)~predict(fit1, Ytraindata))
survConcordance(Surv(Ytestdata$time,Ytestdata$status)~predict(fit1, Ytestdata))

# Model C
library(survival)
library(rms)
```

```
fit1 <- coxph(Surv(time,status)~Age + Ca125 + FIGO + LVSI + P53, data =
Ytraindata)
survConcordance(Surv(Ytraindata$time,Ytraindata$status)~predict(fit1, Ytraindata))
survConcordance(Surv(Ytestdata$time,Ytestdata$status)~predict(fit1, Ytestdata))

# Our model
library(survival)
library(rms)
fit<-coxph(Surv(time, status) ~ Age + FIGO + Grade + LVSI + Ca125 +
ProMisE_molecular +HALP + Adjuvant_treatment, data = Ytraindata)
survConcordance(Surv(Ytraindata$time,Ytraindata$status)~predict(fit,Ytraindata))
survConcordance(Surv(Ytestdata$time,Ytestdata$status)~predict(fit,Ytestdata))
```
